# From Cerebrovascular Injury to Brain Cancer: The Role of Blood–Brain Barrier Dysfunction

**DOI:** 10.3390/biomedicines14030511

**Published:** 2026-02-26

**Authors:** Stanisław Kisiel, Michał Pawlik, Wojciech Jan Niemcewicz, Wincenty Janicki, Julia Świerczyńska, Karolina Romanczuk, Robert Zdanowski, Agata Borkowska

**Affiliations:** 1 Laboratory of Molecular Oncology and Innovative Therapies, Military Institute of Medicine-National Research Institute, Szaserow 128, 04-141 Warsaw, Poland; 2 Faculty of Medicine, University of Warsaw, ul. Żwirki i Wigury 101, 02-089 Warsaw, Poland; 3 Faculty of Medicine, Medical University of Lublin, Al. Racławickie 1, 20-059 Lublin, Poland

**Keywords:** blood–brain barrier, stroke, brain cancer, brain metastasis, tight junctions, neuroinflammation, hypoxia

## Abstract

Stroke and brain cancer are severe disorders of the central nervous system (CNS) associated with high morbidity and mortality. Although each condition has been extensively studied individually, growing evidence suggests that cerebrovascular injury may influence the development of brain malignancies. This narrative review synthesizes current experimental, clinical, and epidemiological data supporting a mechanistic link between stroke and brain cancer, with a particular focus on blood–brain barrier (BBB) dysfunction. We discuss how stroke-induced hypoxia, oxidative stress, and neuroinflammation disrupt BBB integrity, promote endothelial activation, and induce the expression of adhesion molecules that facilitate arrest, extravasation, and survival of circulating tumor cells within the CNS. Additionally, post-stroke regenerative signaling, angiogenesis, and immune modulation may contribute to the formation of a permissive microenvironment that supports tumor initiation and metastatic growth. Available epidemiological studies, clinical observations, and case reports are reviewed to evaluate the strength and limitations of the association between cerebrovascular events and subsequent brain cancer. Although the co-occurrence of stroke and brain cancer remains relatively uncommon, elucidating the shared molecular and cellular mechanisms underlying this relationship can improve risk stratification and allow the development of diagnostic and therapeutic strategies aimed at preserving BBB integrity and reducing long-term oncological complications after stroke.

## 1. Introduction

Advances in medical and biological research have substantially extended human life expectancy; however, this increased longevity has been accompanied by a growing burden of debilitating diseases. Among non-communicable disorders, cerebrovascular accidents—commonly referred to as strokes—remain a leading cause of mortality and long-term disability worldwide, ranking third overall. Epidemiological projections indicate that up to one in four individuals will experience a stroke during their lifetime [1]. Clinically, strokes are broadly classified into ischemic stroke (IS), resulting from cerebral vessel occlusion, and hemorrhagic stroke (HS), characterized by intracerebral or subarachnoid bleeding. In addition, transient ischemic attacks (TIAs) represent episodes of cerebral ischemia without radiological evidence of acute infarction [2]. Despite their heterogeneity, all major stroke subtypes share a common pathophysiological consequence—disruption of the neurovascular unit (NVU) and impairment of blood–brain barrier (BBB) integrity—which can persist beyond the acute phase and influence long-term tissue remodeling.

For clarity, several related but conceptually distinct terms used throughout this review require brief definitions. The BBB denotes the highly specialized endothelial interface that regulates molecular and cellular exchange between the systemic circulation and the healthy central nervous system. Disruption of the BBB refers to the loss of its structural and functional integrity, a phenomenon commonly observed after cerebrovascular injury, including IS and hemorrhagic stroke (HS). In contrast, the blood–tumor barrier (BTB) represents a pathologically remodeled and spatially heterogeneous vascular interface that emerges within primary brain tumors or metastatic lesions and differs from the physiological BBB in permeability, transporter expression, and cellular composition.

Beyond endothelial alterations, neurovascular unit (NVU) dysfunction encompasses coordinated changes across multiple cellular components—including endothelial cells, pericytes, astrocytes, microglia, neurons, and the extracellular matrix—that collectively regulate cerebrovascular stability and brain homeostasis. In the context of stroke and brain cancer, BBB disruption, BTB formation, and NVU dysfunction should be viewed as interconnected but temporally and mechanistically distinct processes that together shape a microenvironment permissive to tumor initiation, progression, and metastatic colonization.

In parallel with cerebrovascular disorders, the incidence of brain tumors is expected to increase with progressive population ageing. Brain tumors may arise within the CNS as primary malignancies, including gliomas and astrocytomas, or develop as secondary lesions through metastasis of peripheral tissues. According to the most recent epidemiological reports, the incidence of brain cancer in the United States is approximately 25.34 per 100,000 individuals. Of these cases, approximately 73% are classified as non-malignant, whereas 27% are malignant [3]. Notably, more than half of malignant tumors correspond to World Health Organization grade IV astrocytomas, namely glioblastoma, which remains associated with a dismal prognosis, with only approximately 25% of patients surviving beyond two years following initiation of treatment [4]. Current standard-of-care therapy combines surgical resection, radiotherapy, and chemotherapy; nevertheless, median overall survival remains limited to approximately 14 months [5]. Given that global mortality rates from brain tumors have remained largely unchanged since 1990, there is a pressing need for novel therapeutic strategies and improved approaches to early diagnosis [6]. Importantly, the poor prognosis of malignant brain tumors is closely linked not only to their intrinsic aggressiveness but also to the presence of the BBB, which acts as both a physical and biological barrier influencing tumor evolution, immune interactions, and therapeutic accessibility.

In recent years, increasing attention has been directed towards the shared molecular mechanisms underlying stroke and brain cancer, raising the possibility of a bidirectional relationship between these conditions [7,8,9,10,11,12,13,14,15,16]. Although previous studies and reviews have described epidemiological associations and overlapping risk factors between cerebrovascular disease and malignancy, these analyses have remained largely descriptive or focused on systemic mechanisms. Notably, the BBB has been discussed primarily either as a victim of cerebrovascular injury or as an obstacle to drug delivery in brain tumors, rather than as an active mechanistic interface dynamically linking post-stroke pathophysiology with tumor initiation and metastatic permissiveness. Consequently, although experimental, clinical, and epidemiological data supporting interactions between stroke and brain cancer are accumulating, an integrative framework that positions BBB dysfunction as a central mediator of this axis remains lacking.

Therefore, this review aims to highlight the BBB as a critical and dynamic mediator of pathogenic processes triggered by cerebrovascular injury and to discuss the molecular and cellular pathways through which stroke-induced BBB dysfunction may contribute to the development of primary and metastatic CNS tumors. We propose that sustained alterations of BBB integrity after stroke can create a tumor-permissive microenvironment, providing mechanistic plausibility for increased vulnerability to brain tumor development or metastatic seeding, while acknowledging that current evidence remains largely associative and hypothesis-generating rather than causally definitive. By integrating mechanistic insights, epidemiological observations, and emerging translational perspectives, this review seeks to delineate unresolved controversies and identify priority areas for future research at the intersection of cerebrovascular disease, BBB biology, and neuro-oncology.

## 2. Blood–Brain Barrier

The BBB is a highly specialized and dynamic interface that regulates molecular exchange between the CNS and the systemic circulation while preserving neural homeostasis. It ensures controlled transport of essential nutrients, including glucose, amino acids, nucleotides, and selected neurotransmitters, while simultaneously protecting neural tissue from toxic metabolites, pathogens, and xenobiotics. Beyond its classical barrier function, the BBB actively participates in metabolic regulation, immune surveillance, and neurovascular signaling.

Structurally, the BBB is formed by non-fenestrated brain microvascular endothelial cells interconnected by complex junctional systems and supported by a specialized basement membrane, pericytes, astrocytic endfeet, vascular smooth muscle cells, and associated immune and glial components of the NVU [17]. Endothelial cells line the cerebral capillaries and are interconnected by tight junctions (TJs), which restrict paracellular diffusion and confer the high electrical resistance characteristic of the barrier. TJs consist of transmembrane proteins, including claudins, occludin, and junctional adhesion molecules (JAMs) [18], which are anchored to the actin cytoskeleton via zona occludens (ZO) proteins [19]. Contrary to earlier assumptions, TJs are not static structures but undergo continuous remodeling in response to physiological stimuli and pathogenic processes [20]. Their stability and organization are further supported by adherent junctions (AJs), primarily composed of E-, N-, and VE-cadherins, which not only contribute to barrier cohesion but also regulate the expression and localization of the TJ components [18]. Disruption or downregulation of these junctional complexes increases permeability of the BBB, with claudin-5 recognized as a key determinant of paracellular tightness in cerebral vessels [15,19,20].

In parallel, BBB endothelial cells express a range of ATP-binding cassette (ABC) transporters, including P-glycoprotein (P-gp), multidrug resistance-associated proteins (MRPs), and breast cancer resistance protein (BCRP), which actively extrude xenobiotics and metabolic waste products back into the bloodstream [19,21]. This efflux system constitutes a critical protective mechanism; however, it also represents a major obstacle to effective CNS drug delivery.

The integrity of the BBB is further reinforced by pericytes embedded within the basement membrane. Together with vascular smooth muscle cells, pericytes regulate cerebral blood flow through contractile activity [22] and contribute to vessel stabilization. Importantly, pericytes play a central role in maintaining the integrity of the BBB, modulating endothelial gene expression, and shaping the local inflammatory environment through the release of cytokines and signaling molecules [23].

Astrocytes form the outer cellular layer of the BBB and exert profound regulatory effects on barrier function. Their terminal processes, known as endfeet, ensheathe brain microvessels and interact closely with endothelial cells and pericytes, providing mechanical support to the basement membrane [23,24,25]. Astrocytes express key channels and transporters, including potassium channels and aquaporin-4 (AQP4), which regulate ion and water homeostasis and thus contribute to the balance of cerebrospinal fluid (CSF) [25]. Moreover, astrocytes can attenuate microglial activation and promote BBB repair under certain conditions, highlighting their role in barrier regeneration [26]. Experimental transplantation studies further demonstrate that astrocytes can induce BBB-like properties in non-CNS vasculature, underscoring their importance in barrier formation and maintenance [27].

Together, endothelial cells, pericytes, astrocytes, junctional complexes, and associated extracellular matrix components constitute the neurovascular unit (NVU)—a functional and clinically relevant entity that integrates vascular, neuronal, and glial signaling [28]. NVU dysfunction is a hallmark of numerous neurological disorders, including ischemic stroke, traumatic brain injury, Alzheimer’s disease, and subarachnoid hemorrhage, all of which are characterized by varying degrees of BBB disruption [29]. Consequently, the BBB should be viewed not merely as a passive barrier but as an active regulator of CNS homeostasis, whose dysregulation represents a central pathogenic event in neurological and neuro-oncological disorders.

## 3. Stroke-Induced Mechanisms of BBB Dysfunction

**Figure 1:**
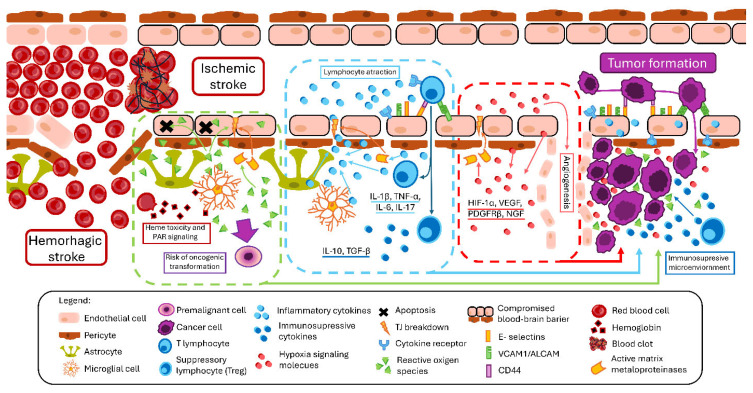
Stroke-induced BBB (blood–brain barrier) disruption and its effects on tumorigenesis. Ischemic stroke (IS) is usually caused by thrombus occluding brain blood vessels. Sudden hypoperfusion triggers the release of reactive oxygen species (ROS) resulting in oxidative stress, leading to apoptosis of endothelial cells. Additionally, ROS-mediated DNA damage can be a driver for oncogenic mutations. In hemorrhagic stroke (HS) oxidative stress plays a more substantial role, as it is further exacerbated by heme toxicity and plasmin signaling pathways. Tissue damage also activates release of inflammatory cytokines that attract immune cells. Activated endothelial cells present adhesive molecules, such as E-selectins, vascular cell adhesion molecule 1 (VCAM-1) and activated leukocyte cell adhesion molecule (ALCAM), allowing lymphocytes and macrophages to enter the central nervous system (CNS). Their presence can either amplify or reduce the immune response. In response to ischemia, hypoxic response mediated by hypoxia inducible factor 1 a (HIF-1a) triggers the release of factors (vascular endothelial growth factor (VEGF), platelet derived growth factor receptor β (PDGFRβ)) that are responsible for cell survival and angiogenesis. All these processes result in disruption of BBB, mediated by activation of matrix metalloproteinases (MMP) and down regulation of endothelial tight junctions. Described processes create a favorable tumor microenvironment, supporting a pre-metastatic niche or allowing primary neoplasms to develop.

Stroke encompasses a heterogeneous group of cerebrovascular events that differ in etiology and clinical presentation but share a common pathological hallmark—disruption of BBB integrity. Whether caused by ischemia, hemorrhage, or transient hypoperfusion, stroke induces a complex cascade of metabolic, inflammatory, and vascular responses that compromise endothelial function and alter neurovascular homeostasis. Increasing evidence indicates that BBB dysfunction not only contributes to acute neuronal injury but may also persist beyond the initial insult, thus shaping long-term neurological outcomes and creating a permissive microenvironment for secondary pathological processes. The exact interplay between the diseases discussed and their molecular mechanisms is summarized in Figure 1.

BBB dysfunction represents a central pathological characteristic in the spectrum of cerebrovascular events, including IS, HS, and TIA. Although these conditions are driven by distinct initiating mechanisms, they converge on endothelial injury, inflammatory activation, and loss of BBB integrity, collectively contributing to acute neuronal damage and long-term neurological deterioration [30].

In IS, oxygen and glucose deprivation initiate a cascade of enzymatic and oxidative processes that progressively compromise vascular integrity. Early after ischemia, increased activity of matrix metalloproteinases (MMP-3, MMP-9, and MMP-12) leads to degradation of key components of the basement membrane and TJ complexes, a process further amplified by pro-inflammatory cytokines such as tumor necrosis factor α (TNF-α) and interleukins (IL’s), including IL-1β, and IL-6, as well as by reactive oxygen species (ROS) [31,32,33]. Consequently, paracellular permeability increases, facilitating rapid influx of peripheral immune cells into the CNS and promoting secondary neuroinflammation [32,34,35]. Beyond structural leakage, ischemia also profoundly alters the transport function of the BBB. Dysregulation of influx and efflux transporters results in the accumulation of toxic metabolites and impaired delivery of neuroprotective substrates, including glucose and antioxidants, thereby reducing neuronal resilience and complicating pharmacological intervention [31].

The loss of BBB integrity further predisposes ischemic tissue to hemorrhagic transformation. Extravasation of blood into the brain parenchyma causes mechanical compression, while hemoglobin, heme, and iron released from lysed erythrocytes act as potent drivers of oxidative stress, lipid peroxidation, and endothelial apoptosis [36]. Thrombin—derived both from vascular leakage and local production by neurons and astrocytes—plays a pivotal role in secondary injury. Through activation of protease-activated receptors (PARs), particularly PAR-1, thrombin induces cytoskeletal remodeling, intracellular calcium influx, and degradation of TJs, thereby amplifying vasogenic edema [36,37]. In parallel, thrombin enhances the release of pro-inflammatory cytokines, stimulates further activation of MMPs, and directly converts IL-1α into its active form, effectively coupling the coagulation cascade with neuroinflammatory signaling pathways [38].

Although transient and non-fatal by definition, TIAs are increasingly recognized as events capable of inducing subtle yet persistent BBB dysfunction. Brief episodes of cerebral hypoperfusion can trigger endothelial activation and low-level oxidative and enzymatic responses resembling those observed in IS. Clinical studies demonstrate that patients with a history of TIA exhibit elevated serum concentrations of platelet-derived growth factor receptor β (PDGFRβ)—a proposed biomarker of pericyte injury and BBB leakage—as well as increased expression of endothelial adhesion molecules, including vascular cell adhesion molecule 1 (VCAM-1) [39] and intercellular adhesion molecule 1 (ICAM-1) [40]. These molecules promote leukocyte adhesion and transmigration, reflecting an early and potentially reversible stage of barrier compromise. Importantly, the same adhesion pathways are exploited during brain metastasis, where they facilitate the docking and extravasation of circulating tumor cells (CTCs), thereby establishing a plausible mechanistic link between cerebrovascular injury and tumor dissemination [41,42,43,44], which requires further validation in dedicated studies.

In summary, the distinct pathophysiological mechanisms underlying IS, HS, and TIA give rise to disease-specific patterns of BBB dysfunction. In IS, hypoxia-driven oxidative stress initiates activation of MMPs, release of inflammatory cytokines, and degradation of TJ proteins of the tight junction, such as claudin-5 and occludin. HS, by contrast, is characterized by an abrupt and profound loss of barrier integrity, followed by cytotoxic effects of extravasated blood components. Hemoglobin, heme, and iron promote oxidative injury, while thrombin-mediated PAR activation and widespread neuroinflammation exacerbate endothelial damage and vasogenic edema. Although TIA does not result in permanent infarction, accumulating evidence indicates that even transient hypoperfusion can induce persistent, low-grade BBB alterations that may contribute to long-term cerebrovascular vulnerability. The key mechanisms underlying BBB disruption across IS, HS, and TIA are summarized in Table 1.

### 3.1. Oxidative Stress

Oxidative stress refers to a pathological condition in which the production of ROS and reactive nitrogen species (RNS) exceeds the antioxidant capacity of the cell, leading to oxidative damage to DNA, lipids, and proteins [45]. Despite their cytotoxic potential, ROS are integral components of normal cellular metabolism and play essential roles in redox signaling. Physiological sources of ROS include oxidative phosphorylation within the mitochondrial electron transport chain, β-oxidation in peroxisomes, and cytochrome P450–mediated metabolism. Under homeostatic conditions, intracellular redox balance is maintained by antioxidant defense systems, including superoxide dismutase (SOD), glutathione peroxidase (GPx), and catalase [46].

Oxidative stress is a well-established driver of tumorigenesis, as excessive DNA oxidation promotes genomic instability and mutagenesis. ROS and RNS can directly interfere with proto-oncogenes, such as cyclin-dependent kinase 4/6 (CDK4/6), and tumor suppressor genes, including tumor protein p53 (TP53) [47]. Concurrently, ROS activate major pro-survival and pro-proliferative signaling pathways—including MAPKs, the Akt/NF-κB axis, mTOR, TGF-β, and AMPK—thereby promoting cellular proliferation, inhibiting apoptosis, and facilitating tumor progression [47,48,49,50,51,52,53]. These pathways are particularly relevant in glioblastoma, where dysregulation of Akt–mTOR signaling is frequently observed due to loss-of-function mutations in PTEN [54].

Additional evidence indicates that ROS act downstream of epidermal growth factor receptor (EGFR) signaling, modulating DNA-dependent protein kinase (DNA-PK) interactions with p53 and leading to functional suppression of p53-mediated apoptosis [48]. ROS have also been implicated in EGFR-driven cytoskeletal remodeling and enhanced motility of GBM cells—processes essential for local invasion and metastatic dissemination [55,56]. Importantly, these tumor-promoting effects of oxidative stress gain particular relevance in the post-stroke brain, where sustained ROS production coincides with compromised BBB integrity and altered neurovascular homeostasis.

Beyond Akt-dependent pathways, ROS activate c-Jun N-terminal kinase (JNK) signaling. JNK activation promotes the proliferation and tumorigenicity of glioma stem cells [57]. At the same time, JNK signaling contributes to BBB disruption by inducing apoptosis in endothelial cells and oligodendrocytes, promoting tight junction degradation, and amplifying neuroinflammatory responses [57]. This dual role of JNK signaling—enhancing tumor aggressiveness while impairing BBB integrity—creates conditions permissive for metastatic spread, as demonstrated in experimental models of non-small cell lung cancer (NSCLC) brain metastasis [58].

In response to oxidative stress, endothelial cells secrete a range of pro-inflammatory cytokines, including TNF-α, IL-1β, IL-6, IL-17, and TGF-β. These mediators induce the expression of endothelial adhesion molecules, such as P- and E-selectins and ICAM-1 [47,59]. Circulating tumor cells can exploit these adhesion pathways to initiate vascular arrest, extravasation, and metastatic colonization of the brain [60,61]. Furthermore, cytokine-mediated activation of nicotinamide adenine dinucleotide phosphate (NADPH) oxidase and xanthine oxidase, along with other signaling pathways [7], establishes a positive feedback loop that sustains oxidative stress within the post-stroke neurovascular microenvironment, thereby further weakening BBB integrity and facilitating CNS invasion by metastatic cells.

Paradoxically, excessive ROS accumulation can also activate apoptotic pathways and induce mitophagy, thereby limiting tumor progression [7,59]. Cellular adaptation to oxidative stress is largely mediated by nuclear factor erythroid 2–related factor 2 (NRF2), which regulates the expression of antioxidant enzymes such as GPx and SOD. While NRF2 activity may exert tumor-suppressive effects under physiological conditions, its sustained activation in cancer has been associated with therapy resistance and poor clinical outcomes, highlighting the context-dependent role of redox signaling in oncology [47,62,63].

In the setting of cerebrovascular injury, oxidative stress represents a primary driver of BBB dysfunction rather than a secondary epiphenomenon. During ischemia–reperfusion injury, reoxygenation triggers excessive production of ROS, including superoxide anions, hydroxyl radicals, hydrogen peroxide, and peroxynitrite [33]. These reactive species compromise BBB integrity through lipid peroxidation, protein oxidation, endothelial apoptosis, and activation of matrix metalloproteinases MMP-2 and MMP-9, leading to degradation of the basement membrane and tight junction complexes and increased BBB permeability [31,34].

Collectively, these oxidative stress–driven mechanisms disrupt the BBB at both cellular and paracellular levels, creating a permissive microenvironment that may support primary tumor initiation as well as metastatic colonization within previously injured brain regions. Key ROS-responsive signaling pathways that link oxidative stress with BBB disruption and glioma progression are summarized in Table 2.

Taken together, oxidative stress emerging after cerebral ischemia or hemorrhage represents a critical driver of BBB dysfunction rather than an isolated byproduct of tissue injury. In the post-stroke setting, excessive reactive oxygen species directly compromise endothelial tight junction integrity, promote endothelial activation, and enhance BBB permeability. These alterations facilitate the adhesion and transmigration of circulating tumor cells and may also support malignant transformation within vulnerable brain regions by sustaining a pro-oxidative, DNA-damaging microenvironment. Thus, oxidative stress constitutes a mechanistic bridge linking acute cerebrovascular injury with long-term tumor-permissive BBB alterations.

### 3.2. Neuroinflammation

Neuroinflammation is an inflammatory response within the CNS that is frequently initiated by oxidative stress and tissue injury [64,65]. A defining feature of this process is the activation of glial cells, which leads to the release of pro-inflammatory cytokines such as TNF-α and multiple interleukins [25,66,67]. These mediators profoundly influence neurovascular homeostasis by modulating endothelial function, immune cell recruitment, and the structural and functional integrity of the BBB.

TNF-α–activated astrocytes engage the signal transducer and activator of transcription 3 (STAT3) signaling pathway, which plays a pivotal role in the post-stroke formation of a tumor-promoting microenvironment by enhancing immunosuppression and compromising BBB integrity. This signaling cascade facilitates tumor cell survival and invasion and contributes to the establishment of a microenvironment favorable for metastatic dissemination. Notably, TNF-α may also be secreted by tumor cells, further amplifying inflammatory signaling within the already vulnerable post-ischemic brain microenvironment [68].

Following IS, microglia rapidly adopt an activated phenotype, reflecting an early and robust inflammatory response. Ju et al. demonstrated that extravasation of blood significantly intensifies microglial activation, underscoring the close relationship between vascular injury and immune signaling [69]. In parallel, endothelial cells, microglia, and astrocytes secrete interferon-induced protein 35 (IFP35) in response to acute ischemia. Through activation of the NF-κB pathway, IFP35 sustains and amplifies inflammatory signaling within NVU [70].

Activation of the RelA/NF-κB axis in microglia has been associated with enhanced metastatic potential in experimental models, including melanoma brain metastasis [71]. In contrast, therapeutic targeting of this pathway can shift microglial polarization toward a more pro-inflammatory and antitumor phenotype. In brain microvascular endothelial cells, NF-κB activation induces downregulation of TJ proteins—particularly claudin-5 and occludin—leading to BBB destabilization [66,72]. Importantly, BBB disruption not only results from neuroinflammation but also perpetuates it, as increased permeability facilitates the entry of plasma proteins and peripheral immune cells into the CNS, thereby reinforcing inflammatory signaling loops and sustaining a tumor-permissive microenvironment [25,66].

Oligodendrocyte precursor cells (OPCs), which are closely associated with brain microvascular endothelial cells [25], contribute to BBB stability through the secretion of TGF-β1. This cytokine promotes the expression of TJ-associated proteins, including ZO-1, occludin, and claudin-5, in endothelial cells. However, OPC migration along blood vessels can displace astrocytic endfeet and disrupt astrocyte–endothelial interactions, as demonstrated in three-dimensional in vitro models [73,74]. Moreover, OPC activity triggers neuroinflammatory signaling, activates microglia, and promotes the recruitment of macrophages and T lymphocytes from the peripheral circulation [74,75], collectively weakening BBB integrity in the post-stroke setting.

Pericytes further modulate BBB responses during post-ischemic neuroinflammation by secreting neurotrophic and pro-angiogenic factors, including BDNF and pleiotrophin (PTN), which support endothelial survival but simultaneously activate NF-κB signaling, angiogenesis, and tumor-associated pathways observed in gliomas [76,77,78,79].

Breakdown of the BBB enables infiltration of circulating immune cells into the CNS, a process tightly regulated by chemokines released from glial and vascular cells. Astrocyte-, microglia-, and pericyte-derived chemokines such as MCP-1, CXCL1, and macrophage inflammatory proteins regulate immune cell recruitment, vascular remodeling, and sustained inflammatory signaling [32,67,80,81,82,83,84,85,86]. Collectively, these chemokine-driven processes amplify neuroinflammation, compromise BBB integrity, and facilitate immune evasion and metastatic colonization within the CNS [23,83,87,88,89,90,91].

The key neuroinflammatory mediators involved in BBB dysfunction and the establishment of a permissive tumor microenvironment following cerebrovascular injury are summarized in Table 3.

In the context of stroke, neuroinflammation extends beyond transient immune activation and becomes a sustained modifier of BBB structure and function. Activated microglia, infiltrating immune cells, and pro-inflammatory cytokines synergistically disrupt endothelial junctions and promote the expression of adhesion molecules on BBB endothelial cells. This inflammatory remodeling enhances vascular permeability and creates a permissive interface for tumor cell arrest, extravasation, and immune evasion within the CNS. Consequently, post-stroke neuroinflammation provides a direct and mechanistically relevant link between cerebrovascular injury, persistent BBB dysfunction, and increased oncological vulnerability of the brain.

Collectively, these findings highlight a self-reinforcing neurovascular network in which interactions among glial and vascular cells drive sustained neuroinflammation and progressive BBB dysfunction. Persistent activation of this inflammatory axis not only exacerbates post-ischemic injury but also actively shapes a tumor-permissive microenvironment that may facilitate tumor invasion and metastatic dissemination within the CNS. Importantly, many of these inflammatory and angiogenic pathways are further potentiated under hypoxic conditions, which represent a convergent driver of BBB dysfunction following cerebrovascular injury.

### 3.3. Hypoxia

Hypoxia, defined as a state of insufficient oxygen availability at the tissue level, represents a central pathophysiological link between cerebrovascular injury and brain cancer. It may arise as a consequence of ischemia following stroke or as a result of high metabolic demand and insufficient vascularization within rapidly growing tumors [107]. Hypoxia-inducible factor 1 (HIF-1) is the main transcriptional regulator of cellular adaptation to reduced oxygen availability. Under normoxic conditions, the HIF-1α subunit undergoes rapid proteasomal degradation, whereas hypoxia stabilizes HIF-1α, allowing its translocation to the nucleus and transcriptional activity. HIF-1α expression can also be enhanced by ROS [26] and by growth factor–dependent signaling pathways involving vascular endothelial growth factor (VEGF), PDGF, and angiopoietins (Ang-1/Ang-2) [95,108], thereby integrating hypoxic and post-stroke inflammatory cues within the neurovascular unit.

Through regulation of more than one hundred target genes, HIF-1α exerts pleiotropic effects that include cell survival, angiogenesis, metabolic reprogramming [103,108], and inflammatory signaling [109]. In the context of IS, HIF-1α plays a dual role in BBB regulation. While certain HIF-1α–dependent pathways confer neuroprotection, others contribute to barrier destabilization. Canonical HIF-1α targets include erythropoietin (EPO) and glucose transporter 1 (GLUT1), which promote oxygen delivery and metabolic support to hypoxic tissues [7]. Notably, these adaptive responses may be co-opted in the post-stroke brain to support tumor cell survival and growth. Downstream HIF-1α targets such as GLUT1/3, VEGF, EPO, and BCL2/adenovirus E1B 19 kDa-interacting protein 3 (BNIP3) enhance glucose uptake, angiogenesis, and metabolic flexibility in cancer cells [7,48]. In parallel, HIF-1α suppresses apoptotic signaling by inhibiting cytochrome c release, poly(ADP-ribose) polymerase (PARP) cleavage, and p53 activation, thereby promoting tumor cell survival [7].

Despite its adaptive functions, HIF-1 signaling has profound disruptive effects on BBB integrity. Experimental BBB models demonstrate that HIF-1α activates downstream pathways such as RhoA/ROCK-1/-2 and myosin light-chain kinase (MLCK), leading to cytoskeletal reorganization and subsequent disassembly of tight junction proteins, including claudin-5 and occludin [110]. Additional mechanisms of BBB disruption involve hypoxia-induced overexpression of matrix MMP-9, which degrades both TJs and components of the basement membrane [96]. Clinical evidence supports these findings: Wu et al. reported that elevated levels of HIF-1α in patients with intracerebral hemorrhage were positively correlated with TNF-α expression and inversely correlated with ZO-1 levels in peripheral blood mononuclear cells, reflecting molecular changes associated with BBB impairment, later confirmed in rat models [107].

In endothelial cells and astrocytes, HIF-1α drives upregulation and secretion of VEGF [109]. VEGF promotes sprouting angiogenesis, a process closely resembling tumor neovascularization. The binding of VEGF to vascular endothelial growth factor receptor 2 (VEGFR2) activates endothelial cells and induces detachment of pericytes from the basal lamina and extracellular matrix [101] or pericyte apoptosis [105]. Concurrently, endothelial cells downregulate the expression of tight junction proteins such as claudin-5 and ZO-1 [106], further compromising barrier integrity. Similar mechanisms have been described in gliomas, where tumor-derived exosomes containing VEGF exacerbate BBB disruption [111]. Moreover, VEGF-induced secretion of MMP-2 and MMP-9 by neurovascular unit cells accelerates degradation of the basement membrane and endothelial TJs [105,107,109]. Collectively, these hypoxia-driven processes converge on BBB destabilization, promote vasogenic edema, and create conditions that are permissive for tumor cell extravasation into the brain parenchyma [112].

**Figure 2:**
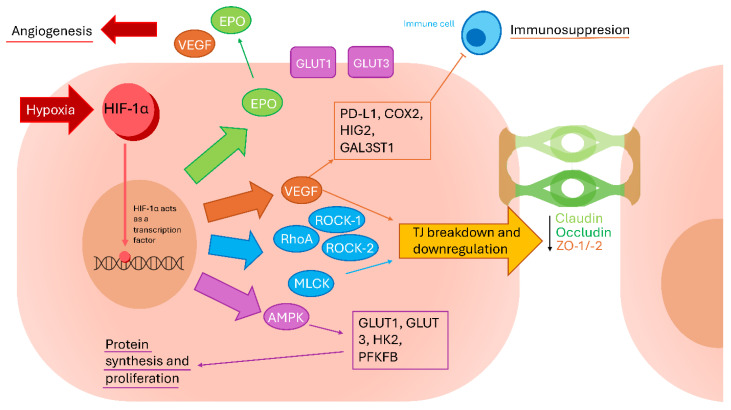
Scheme summarising hypoxic mechanisms responsible for cancer development.

Beyond BBB disruption, post-stroke hypoxia creates a favorable microenvironment for tumor development and progression. HIF signaling is a major driver of GBM pathogenesis. Acting downstream of AMPK, HIF-1α induces the expression of glucose transporters (GLUT1/3), glycolytic enzymes (hexokinase 2, 6-phosphofructo-2-kinase/fructose-2,6-bisphosphatase), and enzymes involved in serine and glycine biosynthesis. Activation of this metabolic program has been correlated with a poor prognosis in patients with GBM [48]. Hypoxia also promotes the establishment of an immunosuppressive tumor microenvironment characterized by the accumulation of regulatory T cells (Tregs), myeloid-derived suppressor cells (MDSCs), and polarization of tumor-associated macrophages toward the M2 phenotype [113,114]. Furthermore, HIF-1α enhances immune evasion by upregulating VEGF/VEGFR signaling and inducing expression of programmed death ligand 1 (PD-L1), thereby impairing T-cell–mediated antitumor responses. Additional HIF-1α–regulated genes, including cyclooxygenase-2 (COX-2), hypoxia-inducible gene 2 (HIG2), and galactosylceramide sulfotransferase 1 (GAL3ST1), further enable cancer cells to escape NK cell–mediated cytotoxicity [115]. Figure 2 portrays the interplay of hypoxia-related signaling pathways.

Although hypoxia-induced responses initially support tissue repair following stroke, these same mechanisms may inadvertently foster a tumor-permissive microenvironment by promoting angiogenesis, increasing nutrient availability, and weakening BBB integrity.

Post-stroke hypoxia represents a potent and sustained stimulus for BBB remodeling with direct relevance to tumor biology. Hypoxia-driven activation of HIF-dependent signaling pathways alters endothelial metabolism, downregulates tight junction proteins, and promotes angiogenic and pro-survival responses within the neurovascular unit. These changes not only compromise BBB integrity but also generate a microenvironment that mirrors key features of the tumor niche, including metabolic reprogramming and immune suppression. As a result, hypoxia serves as a unifying mechanism through which stroke-induced BBB dysfunction may facilitate both primary tumor initiation and metastatic colonization of the brain.

## 4. Stroke-Induced BBB Dysfunction as a Driver of Brain Metastasis

Brain metastases represent the most common intracranial malignancies and affect approximately one in four patients with selected primary cancers, particularly melanoma and lung cancer [116]. Despite their clinical relevance, the mechanisms underlying the organ-specific tropism of CTCs toward the brain remain incompletely understood. The predominant “seed and soil” hypothesis posits that metastatic colonization depends on reciprocal interactions between disseminated tumor cells (“seeds”) and a permissive organ microenvironment (“soil”) [117]. In this context, stroke-induced alterations of the cerebral microvasculature and BBB integrity may critically reshape the brain microenvironment, facilitating metastatic seeding and outgrowth.

To establish secondary tumors within the brain, CTCs must cross the BBB through a multistep process that closely resembles leukocyte transendothelial migration [118]. The initial step involves transient interactions between CTCs and endothelial adhesion molecules, leading to rolling and deceleration of tumor cells along the cerebral microvasculature. This process is mediated primarily by endothelial E- and P-selectins [60,119].

Importantly, IS induces robust endothelial activation characterized by upregulation of selectins, including CD62E, largely driven by inflammatory cytokines such as TNF-α. Elevated expression of E-selectins has been shown to promote brain metastasis of NSCLC [120]. Rai et al. further demonstrated that NSCLC cells release factors—including cystatin C, cathepsin L, VEGF, and TNF-α—that enhance tumor cell adhesion to endothelial E-selectins [121]. Notably, these same mediators are also released following IS [80,122], suggesting that post-stroke inflammatory signaling may actively support metastatic arrest within cerebral vessels.

Following initial tethering, CTCs establish stable adhesion to the endothelium through stronger molecular interactions. Activated leukocyte cell adhesion molecule (ALCAM), expressed by both endothelial cells and tumor cells derived from breast and lung cancers, mediates homophilic binding that arrests CTCs within cerebral microvessels. In parallel, very late antigen-4 (VLA-4; integrin α4β1), expressed by breast cancer and melanoma cells, interacts with VCAM-1 in endothelial cells [60,61]. E-selectins further strengthen adhesion through binding to CD44 on tumor cells. Experimental disruption of these adhesion pathways markedly impairs brain metastasis, underscoring their essential role in tumor cell extravasation [60].

Once firmly adherent, tumor cells initiate transendothelial migration, which requires localized opening of the BBB. This process is facilitated by activation of matrix MMPs, which degrade TJ proteins, as well as by activation of the PI3K/AKT signaling pathway [60]. Stroke-induced BBB dysfunction lowers the threshold for these processes by weakening endothelial junctions and increasing paracellular permeability. An additional mechanism involves EMT, driven by TGF-β1 and specific microRNAs. During EMT, endothelial cells downregulate junctional proteins and acquire a mesenchymal phenotype characterized by expression of fibronectin, β1 integrin, and α-smooth muscle actin (α-SMA), thereby facilitating tumor transmigration.

After extravasation, tumor cells frequently adhere to the abluminal surface of cerebral capillaries, a process termed vascular co-option. This strategy provides immediate access to nutrients and supports subsequent angiogenic remodeling. Key mediators of vascular co-option include L1 cell adhesion molecule (L1CAM) and neuroserpin [123,124].

Astrocytes play a decisive role in determining whether extravasated tumor cells successfully establish metastatic lesions. Through extensive crosstalk with immune and vascular cells, astrocytes can generate a microenvironment that supports tumor growth. Hosonaga et al. demonstrated that astrocytic expression of STAT3 promotes brain metastasis of breast cancer in experimental models and human samples. STAT3-dependent upregulation of PD-L1 and VEGF-A suppresses CD8^+^ cytotoxic T-cell activity, thereby enabling tumor immune evasion [125]. Importantly, astrocytic STAT3 activation is also observed following CNS injury, including IS [126], further linking cerebrovascular insult to metastatic permissiveness.

Astrocytes additionally respond to tumor-derived extracellular vesicles containing regulatory microRNAs. For example, miR-181c alters astrocytic cytoskeletal organization, leading to increased BBB permeability, whereas miR-122 reprograms astrocytic glucose metabolism, preferentially redistributing nutrients to invading tumor cells. Additional astrocyte-mediated mechanisms mediated by astrocytes include deregulation of TJ protein expression and secretion of immunosuppressive and pro-tumorigenic cytokines [127].

**Figure 3:**
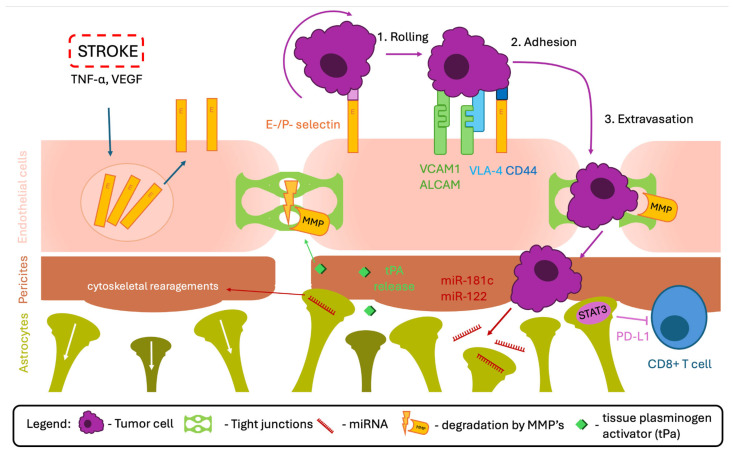
Summary of mechanisms facilitating brain metastasis as a consequence of stroke.

Astrocytes may also exert anti-metastatic effects by releasing plasminogen activators, leading to plasmin activation. Plasmin cleaves Fas ligand (FasL) from astrocytes and L1CAM from tumor cells, thereby inducing tumor cell apoptosis and disrupting adhesion to the basal lamina [124]. However, endogenous plasmin signaling within the CNS can paradoxically promote metastasis. Tissue plasminogen activator activates platelet-derived growth factor-CC (PDGF-CC), enabling its interaction with PDGFR-α on perivascular astrocytes. This signaling cascade induces the expression and release of MMP-2 and MMP-9, which degrade TJ proteins and increase BBB permeability [128], thereby facilitating further tumor cell extravasation (Figure 3).

The structurally and functionally altered vascular interface surrounding brain metastases and angiogenic vessels is known as the blood–tumor barrier (BTB). The BTB exhibits pronounced heterogeneity, characterized by pericyte dysfunction and displacement or loss of astrocytic endfeet, resulting in increased permeability [129]. Although enhanced BTB permeability may facilitate tumor progression by enabling continued tumor cell infiltration, it may also improve delivery of anticancer therapeutics to metastatic lesions [130]. Consequently, therapeutic modulation of the BBB–BTB continuum represents a critical challenge and opportunity in the management of brain metastases.

## 5. Correlation Between Strokes and Brain Cancers

Brain cancers are severe and debilitating conditions, and increasing evidence suggests that their development may share molecular and cellular mechanisms with cerebrovascular injury. Oxidative stress and hypoxia—hallmark features of IS—create a microenvironment conducive to tumor initiation and progression. Stroke-induced endothelial activation promotes expression of adhesion molecules that facilitate attachment of CTCs to the vascular wall, while concomitant BBB disruption lowers resistance to transendothelial migration into the CNS. In later stages, bidirectional crosstalk between tumor cells and astrocytes becomes critical for metastatic outgrowth, as glial cells can suppress antitumor immune responses and provide metabolic support for proliferating cancer cells. Despite these mechanistic overlaps, only a limited number of review articles have addressed the potential correlation between stroke and brain cancer [7], and none have focused specifically on the role of BBB dysfunction in this process.

Elucidating the relationship between stroke and brain cancer is complicated by shared pathophysiological pathways, including inflammation and hypoxia, as well as by temporal challenges. Clinical symptoms of malignancy may precede formal diagnosis by months or even years, making causal inference difficult. Consequently, much of the existing literature has focused on the more established association in which active malignancy increases the risk of stroke [12,15,16]. Brain tumors may precipitate IS through direct vascular compression by expanding tumor masses or through cancer-associated hypercoagulability driven by tumor cells or host immune responses. Moreover, stroke may occur as a complication of oncological treatments, including tumor resection, chemotherapy, and radiotherapy [16]. Brain metastases also represent a recognized risk factor for HS [15], potentially due to BBB disruption during metastatic extravasation.

Beyond molecular mechanisms, stroke-associated neurological deficits may indirectly influence cancer risk by altering lifestyle factors such as physical activity and diet, which have been implicated in cancer development [131]. However, in recent years, growing attention has been directed toward the reverse association—namely, whether cerebrovascular injury may increase the risk of subsequent brain cancer. A limited number of studies provide epidemiological evidence supporting this hypothesis.

A recent meta-analysis reported a hazard ratio of 2.75 for developing brain cancer following cerebrovascular disease [132], indicating a significant epidemiological association. Similarly, a meta-analysis by Rioux et al. demonstrated a higher cumulative incidence of cancer within one year after IS compared to the general population [13]. Notably, most malignancies were diagnosed several months after stroke, suggesting that in many cases, cancer may have been present but clinically undetected at the time of cerebrovascular injury. Long-term observational data further revealed that, ten years after stroke, the incidence of cancer was nearly doubled in patients aged 55 years or younger (17.3% vs. 9.5%), with a weaker but still significant association observed in older cohorts (29.4% vs. 24.9%) [14].

More direct evidence linking stroke with brain cancer development was provided by Chen et al., who analyzed a nationwide Taiwanese cohort of GBM patients [8]. They found that individuals with a prior stroke exhibited a significantly higher adjusted hazard ratio (3.09) for developing brain cancer compared to the general population. Histological analysis revealed strong HIF-1α expression in tumor tissues from stroke-exposed patients, whereas this marker was largely absent in non-exposed controls [8], implicating hypoxia-driven signaling in gliomagenesis. Complementary bioinformatic analysis by Islam et al. identified 57 genes shared between IS and glioblastoma, including CXCR4, a key regulator of brain metastasis [10,133,134]. In contrast, a Mendelian randomization study examining genetic predictors of IS and glioblastoma did not identify a significant causal association [11], suggesting that shared molecular pathways, rather than inherited genetic predisposition, may underlie the observed correlation.

Clinical case reports further support a potential link between stroke and subsequent brain malignancy. Gwak et al. described a 48-year-old male with renal cell carcinoma who developed an acute IS associated with cancer-related coagulopathy, followed by the appearance of brain metastasis eight months later within the infarcted region [135]. Similarly, another report documented brain metastasis from pulmonary adenocarcinoma arising five months after an ischemic stroke caused by occlusion of the middle cerebral artery, with both lesions localized to the same brain territory [136]. In both cases, the authors proposed stroke-mediated BBB disruption as a key etiological factor. Additional hypotheses include tumor exploitation of post-ischemic angiogenesis and creation of a permissive “soil” within infarcted tissue.

Notably, this phenomenon does not appear to be limited to IS. A case study described a 22-year-old female who developed HS in the left frontotemporoparietal region and was diagnosed 16 years later with malignant melanoma metastasis localized to the same area [137]. These observations suggest that brain metastasis may occur independently of stroke subtype, etiology, or latency period.

While individual case reports provide compelling proof-of-concept evidence, their inherent limitations necessitate validation in larger patient cohorts. To date, only one retrospective study has systematically examined this association. In a cohort of 307 patients with non-small cell lung cancer, cerebral infarction emerged as an independent risk factor for brain metastasis (odds ratio 3.303; 95% confidence interval 1.437–7.593; *p* = 0.005) [138]. These findings underscore the need for prospective, hypothesis-driven studies specifically designed to capture temporal dynamics, stroke characteristics, and BBB integrity.

In addition to secondary brain tumors, several reports describe glioblastoma developing within regions previously affected by stroke. We identified three cases of GBM arising after ischemic infarction [139,140,141] and one case following HS [142]. Tumor diagnosis occurred between seven months and three years after the cerebrovascular event, with no radiological evidence of pre-existing malignancy. Collectively, these observations support the concept that ischemia-induced tissue damage, revascularization, and persistent BBB remodeling may create a permissive niche for malignant transformation.

Based on the available evidence, female sex and age between 40 and 60 years appear to represent potential risk modifiers for brain cancer development after stroke [8]. Identification of such subgroups may have clinical relevance, as earlier diagnosis of brain tumors is associated with improved prognosis [143]. However, larger and more diverse cohorts are required to validate these observations and identify additional predisposing factors.

From a translational perspective, therapeutic strategies aimed at preserving BBB integrity may offer dual benefits by mitigating neurodegenerative sequelae of stroke while potentially reducing susceptibility to brain cancer. Several candidate approaches have been proposed based on preclinical evidence, including borneol [144], c-Jun N-terminal kinase (JNK) inhibitors [57], modulation of atractylon-mediated sirtuin 3 (SIRT3) signaling [145,146,147], and Z-ligustilide (Z-Lig). Borneol exhibits anti-inflammatory properties that may attenuate stroke-induced MMP-9 activation and reduce the immunosuppressive potential of gliomas [144]. Similarly, JNK inhibition has been shown to preserve BBB integrity in experimental models of both stroke and cancer [57]. Modulation of SIRT3 signaling stabilizes the BBB through inhibition of the HIF-1α/VEGF axis while simultaneously sensitizing glioblastoma cells to ferroptosis and autophagy, resulting in reduced tumor growth in preclinical studies [145,146,147]. Z-ligustilide, an active compound derived from Ligusticum chuanxiong, reduces neuroinflammation after stroke and improves temozolomide pharmacokinetics in experimental glioblastoma models. Although its precise mechanisms remain incompletely defined, Z-Lig has been shown to modulate hypoxia signaling and downregulate P-glycoprotein, claudin-5, and occludin expression, thereby facilitating drug penetration across the BBB [148,149]. Importantly, the potential adverse consequences of BBB modulation, including excessive barrier permeability, require careful evaluation. At present, all therapeutic strategies discussed remain at the preclinical stage, with supporting evidence derived exclusively from in vitro and animal studies [57,144,145,146,147,148,149]. Accordingly, any potential clinical benefit should be interpreted as prospective rather than established.

Beyond therapeutic approaches, emerging diagnostic strategies aim to detect BBB alterations at early stages. Dynamic contrast-enhanced magnetic resonance imaging (DCE-MRI) is currently a standard technique for in vivo assessment of BBB permeability [150]. Immuno-MRI enables visualization of endothelial activation and adhesion molecule expression associated with neuroinflammation [40]. Other approaches, such as total-body positron emission tomography (PET), allow functional monitoring of BBB permeability across different particle sizes [151]. In parallel, circulating biomarkers of NVU injury—including PDGFRβ, HIF-1α, TNF-α, and ZO-1—can be quantified in peripheral blood, offering promising avenues for early diagnosis and longitudinal monitoring [39,107].

Despite growing mechanistic and epidemiological interest in the relationship between cerebrovascular injury, BBB dysfunction, and brain cancer, this field faces substantial inherent limitations. Stroke is a life-threatening condition in which clinical priorities are necessarily focused on acute, life-saving interventions rather than long-term surveillance or prophylaxis of secondary complications. In addition, the co-occurrence of stroke and brain cancer appears to be relatively rare, as reflected by the limited number of reported clinical cases. Together, these factors constrain both the quality and quantity of available clinical data and complicate the design of systematic, hypothesis-driven studies addressing the stroke–BBB–brain cancer axis.

Moreover, the novelty of current research efforts—largely centered on molecular and cellular mechanisms linking stroke and cancer—further limits the availability of robust clinical evidence. In this context, careful literature selection and integrative analysis are essential for advancing the field, as emphasized by De Simone et al. [152]. Accordingly, given the scarcity of directly relevant clinical studies available as of 2026, the selection of sources included in this review was adapted to reflect the emerging and exploratory nature of this research area.

It is also important to acknowledge several confounding factors that may influence the reported association between stroke and subsequent brain cancer. Both conditions share multiple vascular and lifestyle-related risk factors, including hypertension, diabetes, active smoking, and excessive alcohol consumption, which may partially account for observed correlations [153]. In addition, stroke patients typically receive more frequent and comprehensive medical follow-up, including repeated neuroimaging, which increases the likelihood of detecting developing or previously unrecognized brain tumors compared to the general population, thereby introducing surveillance bias. Furthermore, the presence of occult or asymptomatic brain tumors at the time of the cerebrovascular event cannot be excluded, raising the possibility of reverse causation in a subset of epidemiological analyses. These limitations underscore the need for well-designed, unbiased cohort studies—particularly those comparing the incidence of brain tumors with other cancer types in stroke survivors—to more accurately define the temporal relationship and mitigate detection bias. Nevertheless, the evidence summarized herein highlights BBB dysfunction as a biologically plausible mechanistic link between cerebrovascular injury and brain malignancy. Targeting BBB integrity may therefore represent a promising strategy not only for understanding these rare comorbidities but also for improving outcomes in stroke and brain cancer as independent disease entities.

## 6. Conclusions

The body of evidence reviewed here supports the concept that disruption of the BBB after stroke may contribute to the subsequent development of brain tumors. Epidemiological studies consistently indicate that cerebrovascular events constitute a significant risk factor for brain cancer, a relationship further reinforced by multiple clinical case reports documenting tumor emergence within previously infarcted or hemorrhagic brain regions. Although precise causal mechanisms remain incompletely defined, converging data implicate hypoxia, oxidative stress, and neuroinflammation as central drivers of post-stroke microenvironmental remodeling.

A key contribution of this review is the integration of BBB dysfunction into the stroke–brain cancer axis as a unifying mechanistic framework, rather than viewing BBB alterations solely as secondary consequences of cerebrovascular injury or as barriers to therapeutic delivery. These processes promote degradation of endothelial tight junctions, endothelial activation, and increased expression of adhesion molecules, collectively facilitating adhesion, extravasation, and survival of circulating tumor cells within the central nervous system. In later stages of stroke recovery, growth factor signaling, angiogenesis, metabolic reprogramming, and immune suppression may further support tumor initiation and progression by creating a permissive niche for malignant transformation and metastatic colonization.

Importantly, while current evidence supports strong mechanistic plausibility linking stroke-induced BBB dysfunction with brain tumor development, this relationship should be interpreted as largely associative and hypothesis-generating rather than causally definitive. Based on the data summarized herein, several testable hypotheses emerge, including the existence of a defined temporal window of increased oncological vulnerability following stroke, the prognostic value of persistent BBB permeability changes, and the contribution of specific neuroinflammatory signaling pathways to tumor-permissive BBB remodeling.

Accordingly, future research should prioritize longitudinal, prospective clinical studies that correlate temporal changes in BBB permeability with brain tumor incidence and metastatic burden following cerebrovascular injury. Mechanistically informed in vitro and in vivo models are also needed to dissect shared molecular signatures of BBB disruption, stroke recovery, and tumor progression. Furthermore, interventional approaches targeting BBB-stabilizing and anti-inflammatory pathways, including modulation of NF-κB signaling, warrant systematic evaluation as potential strategies to reduce secondary CNS malignancies or metastatic seeding after stroke.

From a clinical perspective, these findings suggest that enhanced vigilance regarding BBB integrity may be particularly relevant for stroke survivors with a history of malignancies characterized by a high propensity for CNS metastasis, such as lung cancer, breast cancer, and melanoma. In this context, BBB integrity may serve not only as a mechanistic marker but also as a risk stratification tool for identifying high-risk patient subgroups.

There are also diagnostic approaches with potential relevance for clinical translation. Plasma circulating tumor DNA represents a viable diagnostic tool and an alternative to cerebrospinal fluid analysis for detecting brain metastases, offering a minimally invasive option for high-risk patients [154]. In parallel, dynamic susceptibility contrast MRI enables mapping of BBB leakage to identify tissue at risk of infarction and potential metastatic niches [155].

Collectively, these insights position the BBB not only as a passive victim of cerebrovascular injury but as an active, dynamic, and clinically actionable determinant of long-term neurological and oncological outcomes, highlighting BBB integrity as a promising and conceptually unifying target for future diagnostic, prognostic, and therapeutic strategies at the intersection of stroke and brain cancer.

## Figures and Tables

**Table 1:** Collection of BBB degradation mechanisms in different types of strokes. Meaning of symbols: “+”—mechanism leads to disruption of BBB, “−”—mechanism not present/does not influence BBB permeability.

Mechanism of BBB Disruption	Ischemic Stroke	Hemorrhagic Stroke	Transient Ischemic Attack	References
TJ protein degradation (claudin-5, occludin, ZO-1)	+	+	+	[31,33,34]
MMP activation (MMP-9, MMP-2, MMP-12, MMP-3)	+	+	+	[31,33]
Oxidative stress (ROS, lipid peroxidation)	+	+	+	[31,34]
Inflammatory cytokines (TNF-α, IL-1β, IL-6, IL-17)	+	+	+	[31,32]
Microglial and astrocyte activation	+	+	+	[31,32]
Thrombin and PAR signaling	+	+	−	[37]
Neutrophil and lymphocyte infiltration	+	+	−	[32,35]
Pericyte and endothelial injury	+	+	+	[31,34]
BBB transporter dysfunction (P-gp, BCRP, MRPs)	+	−	−	[31]
Mechanical compression	−	+	−	[36]
Mechanism of BBB disruption	Ischemic stroke	Hemorrhagic stroke	Transient ischemic attack	References
TJ protein degradation (claudin-5, occludin, ZO-1)	+	+	+	[31,33,34]
MMP activation (MMP-9, MMP-2, MMP-12, MMP-3)	+	+	+	[31,33]

**Table 2:** Summary of ROS-activated pathways responsible for the growth of primary and metastatic brain tumors.

Pathway/Axis	Mechanism	Outcomes	Sources
p53 inactivation/mutation	Loss of cell cycle control and inhibition of apoptosis	Promotes GBM development	[47,49,62]
EGFR	Inhibition of p53 signaling, regulation of cytoskeletal dynamics and cell motility	Promotes GBM development and invasive behavior	[49,55,56]
Akt/p53/Bcl-2/caspase-9	Inhibition of apoptotic signaling pathways	Promotes tumor cell survival	[62]
Akt/NF-κB	Induction of pro-inflammatory cytokine release and regulation of cell motility	BBB damage facilitating brain metastasis	[34,51,62]
Akt/mTORC1	Cell cycle progression, enhanced protein synthesis, and metabolic reprogramming	Promotes GBM growth, angiogenesis, and energy storage	[54,55]
TGF-β/NOX4	ROS-dependent induction of glioma stem cell (GSC) proliferation and colony formation	Promotes GSC stemness	[31]
AMPK/HIF-1α	Upregulation of glycolytic and serine biosynthesis enzymes	Promotes GBM survival under metabolic stress	[29]
MKK4/JNK	Induction of GSC proliferation and stemness; release of MMP-9, TNF-α, and caspase-9 in BBB cells	Promotes GSC stemness and BBB damage, facilitating brain metastasis	[36,57]
β-catenin	Transcriptional activation of stemness- and proliferation-related genes	Promotes GSC development and maintenance	[53]
NRF2	Upregulation of antioxidant enzymes maintaining ROS below apoptotic threshold	Tumor survival and therapy resistance	[28,42]

**Table 3:** Neuroinflammatory mediators contributing to BBB dysfunction and permissive tumor microenvironment following cerebrovascular injury.

Molecule	Primary Function in Neuroinflammation and BBB Regulation	Cellular Origin	References
Tumor necrosis factor-α (TNF-α)	Amplifies local inflammation; promotes tight junction degradation and BBB destabilization; activates STAT3 signaling	Astrocytes, microglia	[92,93,94,95,96]
Interleukins (IL-1β, IL-6, IL-9, IL-10, IL-12, IL-13, IL-17)	Amplify neuroinflammatory signaling and indirectly increase BBB permeability	Astrocytes, microglia, pericytes	[92,93,94,95]
Nuclear factor κB (NF-κB)	Sustains inflammatory gene expression; downregulates tight junction proteins (claudin-5, occludin)	Endothelial cells, microglia	[81,93,96]
Signal transducer and activator of transcription 3 (STAT3)	Shapes an immunosuppressive and pro-tumorigenic microenvironment; contributes to BBB dysfunction	Astrocytes	[68,97]
Interferon-induced protein 35 (IFP35)	Activates NF-κB signaling and amplifies post-ischemic neuroinflammation	Endothelial cells, microglia, astrocytes	[70]
Transforming growth factor-β1 (TGF-β1)	Enhances the expression of tight junction proteins (ZO-1, occludin, claudin-5)	OPCs	[73]
Platelet-derived growth factor-B (PDGF-B)	Regulates pericyte migration to sites of vascular injury	Endothelial cells	[23]
Brain-derived neurotrophic factor (BDNF)	Supports endothelial survival; enhances tumor-associated signaling via NF-κB activation	Pericytes	[76,83]
Nerve growth factor (NGF)	Supports neuronal and endothelial cell survival during post-ischemic inflammation	Microglia, pericytes	[83,90,98,99]
Neurotrophin-3 (NT-3)	Supports endothelial cell survival and vascular stability	Pericytes	[83]
Pleiotrophin (PTN)	Promotes angiogenic signaling; activates macrophages and microglia; supports tumor growth	Pericytes	[79,83,89]
Monocyte chemoattractant protein-1 (CCL2/MCP-1)	Activates microglia; stimulates astrocytic cytokine release; recruits monocytes/macrophages	Astrocytes, microglia, pericytes	[99,100,101,102]
CCL3 (MIP-1α)	Promotes inflammatory signaling via NF-κB activation	Pericytes	[95,96]
CCL4 (MIP-1β)	Recruits macrophages and microglia	Pericytes	[95,103]
CCL5	Recruits and activates T lymphocytes	Pericytes	[23,87]
CCL11	Attracts eosinophils and modulates immune infiltration	Pericytes	[83,89]
CCL17	Recruits lymphocytes; facilitates dendritic cell transmigration across the BBB; alters microglial morphology	Pericytes	[83,89]
CXCL1	Recruits neutrophils; promotes inflammatory signaling and vascular remodeling	Astrocytes, pericytes	[101,102,104]
CXCL5	Recruits innate and adaptive immune cells; contributes to angiogenic and pro-tumorigenic signaling	Pericytes	[101,105,106]

## Data Availability

No new data were created or analyzed in this study. Data sharing is not applicable to this article.

## Abbreviations

The following abbreviations are used in this manuscript:

ABC	ATP binding cassette
AJ	adherent junction
Akt/NF-κB	kinase Akt mediated nuclear factor κB signaling pathway
ALCAM	activated leukocyte cell adhesion molecule
AMPK	AMP-activated protein kinase
Ang-1	angiopoietin-1
Ang-2	angiopoietin-2
AQP4	aquaporin 4
BBB	blood–brain barrier
BCRP	breast cancer resistance protein
BDNF	brain-derived neurotrophic factor
BNIP3	BCL2/adenovirus E1B 19 kDa—interacting protein 3
BTB	blood–tumor barrier
CCL	C-C motif chemokine ligand
CDK	cyclin-dependent kinase
CNS	central nervous system
COX-2	cyclooxygenase-2
CSF	cerebrospinal fluid
CTC	circulating tumor cell
CXCL1	C-X-C motif chemokine ligand 1
DCE-MRI	dynamic contrast enhanced magnetic resonance imaging
DNA-PK	PK-DNA dependent protein kinase
EGFR	epidermal growth factor receptor
EMT	epithelial to mesenchymal transition
EndMT	endothelial-to-mesenchymal transition
EPO	erythropoietin
GAL3ST1	galactosylceramide sulfotransferase 1
GBM	glioblastoma multiforme
GLUT1	glucose transporter 1
GPx	glutathione peroxidase
HIF-1	Hypoxia-inducible factor 1
HIG2	hypoxia-inducible gene 2
HS	hemorrhagic stroke
I/R	ischemia–reperfusion
ICAM-1	intercellular adhesion molecule 1
IFP35	interferon-induced protein 35
IL	interleukin
IP-10	interferon-γ–inducible protein-10
IS	ischemic stroke
JAM	junctional adhesion molecule
JNK	c-Jun N-terminal kinase
L1CAM	L1 cell adhesion molecule
MAPK	mitogen-activated protein kinase
MCP-1	monocyte chemoattractant protein-1
MDSC	myeloid-derived suppressor cell
MIP-1α	Macrophage Inflammatory Protein 1α
MKK4	mitogen-activated protein kinase 4
MLCK	myosin light-chain kinase
MMP	matrix metalloproteinase
MRP	multidrug resistance associated protein
mTOR	mammalian target of rapamycin
NADPH	nicotinamide adenine dinucleotide phosphate
NF-κB	nuclear factor κB
NGF	nerve growth factor
NK	natural killers
NRF2	nuclear factor erythroid 2–related factor 2
NSCLC	non-small cell lung cancer
NT-3	neurotrophin-3
NVU	neurovascular unit
OPC	oligodendrocyte precursor cell
PAR	protease-activated receptor
PARP	poly(ADP-ribose) polymerase
PAs	plasminogen activators
PDGF	platelet-derived growth factor
PDGF-CC	platelet-derived growth factor-CC
PDGFRβ	platelet-derived growth factor receptor β
PD-L1	programmed death ligand 1
PET	positron emission tomography
P-gp	P-glycoprotein
PI3K	phosphatidylinositol 3-kinase
PKC	protein kinase C
PLC-γ	phospholipase C gamma
PTEN	phosphatase and tensin homolog
PTN	pericytes release pleiotrophin
RNS	reactive nitrogen species
ROS	reactive oxygen species
SIRT3	atractylon-mediated sirtuin 3
SOD	superoxide dismutase
STAT3	signal transducer and activator of transcription 3
TGF-β	transforming growth factor β
TIAs	transient ischemic attacks
TJs	tight junctions
TNF-α	tumor necrosis factor α
TP53	tumour protein p53
Treg	regulatory T cell
VCAM-1	vascular cell adhesion molecule 1
VEGF	vascular endothelial growth factor
VEGFR2	vascular endothelial growth factor receptor 2
VLA-4	very late antigen-4
Z-Lig	Z-ligustilide
ZO	zona occludens
α-SMA	α-smooth muscle actin
